# Disrupted bone microenvironment and immune recovery following total body irradiation in a murine model

**DOI:** 10.1038/s41419-025-08303-7

**Published:** 2025-12-13

**Authors:** Tibor Sághy, Priti Gupta, Karin Horkeby, Petra Henning, Claes Ohlsson, Carmen Corciulo, Marie K. Lagerquist, Andrei S. Chagin, Cecilia Engdahl

**Affiliations:** 1 Department of Rheumatology and Inflammation Research, Sahlgrenska Academy, University of Gothenburg, Gothenburg, Sweden; 2 Department of Internal Medicine and Clinical Nutrition, Sahlgrenska Osteoporosis Centre and Centre for Bone and Arthritis Research, Institute of Medicine, Sahlgrenska Academy, University of Gothenburg, Gothenburg, Sweden; 3 Department of Pharmacology, Institute of Neuroscience and Physiology, Sahlgrenska Academy, University of Gothenburg, Gothenburg, Sweden

**Keywords:** Molecular biology, Experimental models of disease, Translational research, Cell death, Cell biology

## Abstract

Irradiation is an effective therapy for eliminating cancer cells and serves as a critical preparative regimen for hematopoietic stem cell transplantation (HSCT). However, irradiation affects healthy tissue, disrupting bone tissue and bone marrow homeostasis, which leads to skeletal and immune dysfunction. To investigate these effects, we used a female murine model to explore the mechanisms underlying the skeletal damage caused by total body irradiation. Experiments were carried out over a 12-week period of total body irradiation and HSCT, supplemented by an acute study involving only total body irradiation and osteoclastogenesis. Irradiation resulted in a depletion of bone marrow immune cells, followed by an increase in bone marrow cellularity 12 weeks after irradiation and HSCT compared to naive controls, indicating late-stage local immune induction. Cortical and trabecular bone loss appeared 2 weeks post-irradiation and HSCT and persisted throughout the study. This bone damage was accompanied by a sustained increase in apoptotic cells in the bone marrow within 6 h post-irradiation and persisted for up to 12 weeks after irradiation and HSCT. This was coupled with elevated local expression of the pro-apoptotic *Bax* gene and an increase in *TGF-β1*, a gene associated with the clearance of apoptotic cells. In vitro studies demonstrated that macrophages and pre-osteoclasts, but not fully differentiated osteoclasts, efficiently cleared apoptotic cells, resulting in increased levels of TGF-β1 in the culture supernatant. While the clearance of apoptotic cells and associated TGF-β1 signaling were evident, their direct role in skeletal outcomes remains unclear. These findings suggest that persistent apoptotic cells contribute to impaired bone remodeling, potentially affecting osteoclast function and the bone microenvironment. Additional research is needed to investigate whether targeting apoptotic cell clearance can mitigate bone damage and promote skeletal recovery following irradiation.

## Introduction

Irradiation therapy remains a fundamental component in managing various cancers, with more than half of all cancer patients receiving radiotherapy [1]. Despite its effectiveness in reducing tumor size and eradicating hematopoietic cells before hematopoietic stem cell transplantation (HSCT), concerns exist regarding its impact on healthy surrounding tissues, which leads to both immediate and long-term adverse effects [2]. Skeletal tissue is particularly vulnerable to radiation due to its high calcium content [3, 4]. Irradiation, whether total or limited, reduces bone mineral density and disrupts bone microarchitecture [5–7], yet the timeline and underlying mechanisms remain unclear. This weakened bone tissue significantly increases the risk of fractures [7, 8], contributing to higher morbidity and mortality among cancer patients and posing a substantial challenge to overall patient outcomes [9].

Bone marrow hematopoietic stem cells (HSCs), which are essential for regenerating mature blood cells, are highly susceptible to radiation. Even low doses of total body irradiation (TBI) can drastically lower lymphocyte counts, while high doses destroy both mature lymphocytes and HSCs, necessitating HSCT for long-term survival [10]. Irradiation triggers extensive cellular damage through oxidative stress and DNA damage, leading to massive apoptosis. The clearance of apoptotic cells, i.e., efferocytosis, is primarily performed by professional phagocytes, such as macrophages and dendritic cells [11–13]. The maturation of these cells is compromised after irradiation, potentially impacting their efferocytosis capacity.

Osteoclasts originate from monocytes [14]. Monocytes exhibit relative radiation resistance compared to lymphocytes, but, like other immune cells, their numbers decrease after radiation [15]. Since monocytes serve as precursor cells for osteoclasts, this reduction limits osteoclast formation and temporarily impairs bone resorption. However, as monocyte populations recover, this increase can lead to a surge in bone resorption during the later stages of recovery from irradiation [16]. Like other cells that originate from monocytes, osteoclasts can phagocytose pathogens and act as antigen-presenting cells in the bone marrow [17, 18]. Apoptotic osteoblasts and bone lining could stimulate osteoclasts during normal bone remodeling [19–22]. However, the mechanisms by which apoptotic cells regulate osteoclast activity and whether a massive induction of apoptotic cells would influence osteoclast function remain unclear.

Moreover, radiation exposure initiates an inflammatory response within the bone microenvironment [23]. Inflammation triggers the local induction of pro-inflammatory cytokines [24] and transforming growth factor-beta (TGF-β1) [25, 26]. These mediators can affect bone hemostasis and regulate osteoclast activity and differentiation [14, 27, 28].

Radiation-induced damage also leads to the dysregulation of mesenchymal stem cells (MSCs). It shifts MSC differentiation toward adipogenesis and leads to increased marrow adiposity, thereby reducing the ability to form osteoblasts, which collectively impairs bone regeneration [29, 30].

We hypothesize that apoptotic cells regulate the bone microenvironment and modulate bone homeostasis. Therefore, our study aims to characterize changes in immune cells, skeletal tissues, and the role of apoptotic cells in osteoclastogenesis in a mouse model. By elucidating these interactions, we aim to uncover the mechanisms underlying the disruption of bone homeostasis post-irradiation.

## Materials and methods

### Mice

Female C57BL/6 wild-type mice at 8 weeks old were obtained from Janvier Labs (Le Genest Saint Isle, France) and maintained at the Sahlgrenska Academy, Laboratory of Experimental Biomedicine in Gothenburg. In all experiments, the control and irradiated mice that received hematopoietic stem cell transplants (HSCT) were siblings and had similar weights before inclusion. The mice were co-housed in standard cages, kept at 22 °C, and exposed to a 12-h light-dark cycle. They had free access to Teklad Diet 2016 (Envigo, Indianapolis, IN, USA) and tap water. Starting 1 week before irradiation and continuing until 2 weeks post-irradiation, both control and irradiated mice received Enrofloxacin (0.6 mg/ml, Elanco Denmark ApS) in their drinking water as antibiotic treatment. Before euthanasia, the final body mass of the mice was recorded. Mice were anesthetized with Ketador/Dexdomitor (Richter Pharma/Orion Pharma), bled from the axillary vein, and euthanasia was performed via cervical dislocation. Organ weights were determined and normalized to body weight for analysis. Tissue samples were snap-frozen in liquid nitrogen and stored at −80 °C, while bones were fixed in 4% paraformaldehyde (PFA) for 48 h, followed by 70% ethanol, and then stored at room temperature until subsequent analysis. The animal procedures complied with the guidelines of the Animal Ethics Committee of Gothenburg (3230–2020).

### Irradiation

**Fig. 1:**
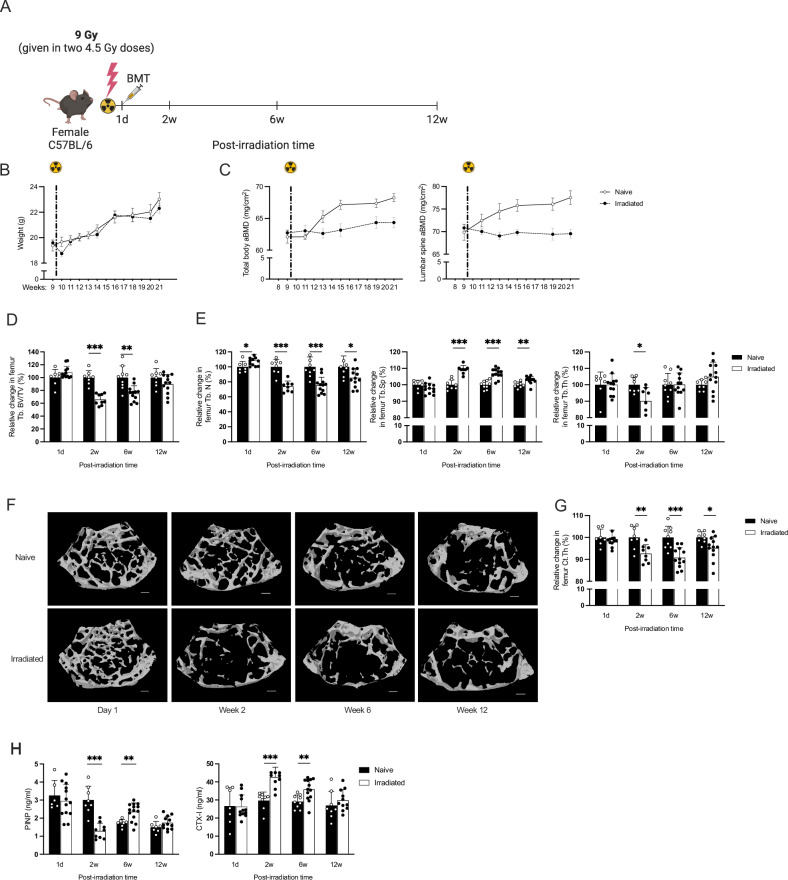
Longitudinal analysis of bone parameters, microstructure, and bone markers following irradiation and hematopoietic stem cell transplantation (HSCT). **A** Schematic diagram showing experiment design of the long-term experiment. Irradiation was performed in 9-week-old mice that were euthanized after an additional 1 day (1d), 2-, 6-, or 12 weeks [12w]. **B** Changes in body weight over the 12-week experiment post-irradiation. A significant difference was observed using the Student’s *t* test 1 week after irradiation and HSCT, at 10 weeks of age. The dotted line indicates the time of irradiation and HSCT. **C** Total body areal bone mineral density (aBMD), and lumbar spine vertebrae (LS) aBMD in irradiated and naive mice. A significant difference was observed with the Student’s *t* test 4 weeks after irradiation and HSCT, and this difference persisted throughout the entire 12-week period in mice aged 13–21 weeks. **D** Micro-computed tomography (μCT) analysis of trabecular bone volume per total volume (Tb. BV/TV) in the femur, shown as relative change versus naive mice, which is set to 100%. **E** Relative changes compared to naive mice (set to 100%) of trabecular number (Tb.N), trabecular separation (Tb.Sp), and trabecular thickness (Tb.Th) in the femur. **F** Representative images of trabecular bone in the femur. The scale bar represents 200 μm. **G** μCT analyses of cortical thickness (Ct.Th) in femur, shown as relative change versus naive mice, which is set to 100%. **H** Serum levels of C-terminal telopeptide (CTX, a bone resorption marker) and procollagen type I N-terminal propeptide (PINP, a bone formation marker). Statistical differences between irradiated and HSCT compared to naive mice at each time point were evaluated using Student’s *t* test. Sample sizes ranged from *n* = 6–12. Data are presented as mean ± SD. Significance levels are indicated as *p < 0.05, **p < 0.01, ***p < 0.001.

For the long-term post-irradiation experiments, followed by HCST performed the same day (a detailed visual irradiation scheme is shown in Fig. 1A), a lethal dose of 9 Gy of total body X-ray irradiation was administered to the mice in two fractions of 4.5 Gy each, with a 4-h interval between the fractions. Mice were euthanized, and bones were collected 1 day, 2 weeks, 6 weeks, and 12 weeks post-irradiation to display the long-term effects of radiation. For the 48-h acute post-irradiation experiment, a single fraction of 9 Gy total body X-ray irradiation was administered (a detailed visual irradiation scheme is shown in Fig. 4A). To display the acute effect of radiation, mice were euthanized, and bones were collected 6-, 12-, 24-, and 48-h post-irradiation.

Irradiation was performed using an RS2000 irradiator with a 0.3 mm copper filter and X-ray tube settings of 160 kV and 25 mA (1.59 Gy/min) (Rad Source Technologies, Buford, GA, USA).

### Hematopoietic stem cell transplantation (HSCT)

For the long-term experiment, bone marrow cells were harvested from sibling donor mice using the Negative Selection EasySep Mouse Hematopoietic Progenitor Cell Isolation Kit (StemCell), following the manufacturer’s instructions. A total of 800,000 enriched HSCs were resuspended in PBS and intravenously injected into the tail vein of irradiated recipient mice, while non-irradiated naive mice received PBS. Our previous study demonstrated successful allogeneic transplantation in the bone marrow and spleen 6 weeks after irradiation, using a similar approach [31].

### Dual-energy X-ray absorptiometry (DXA)

Dual-energy X-ray absorptiometry analysis was conducted several times on the same mice before and after irradiation to evaluate total body areal bone mineral density (aBMD) and lumbar spine (L3–L6) aBMD, using Faxitron UltraFocus DXA (Faxitron Bioptics, LLC, Tucson, AZ, USA) at 40 kV and 0.28 mA for 2.53 s, with a spatial resolution of 24 μm and a 2x geometric magnification.

### High-resolution microcomputed tomography (μCT)

High-resolution microcomputed tomography was performed on the femur and lumbar vertebrae (L5) from the euthanized long-term post-irradiated and naive mice using a Skyscan 1172 scanner (Bruker MicroCT, Aartselaar, Belgium). The lumbar vertebra and femur were imaged with an X-ray tube voltage of 50 kV and a current of 201 μA, using a 0.5 mm aluminum filter. The scanning angular rotation was 180°, and the angular increment was 0.70°. NRecon (version 1.6.9.8, Bruker MicroCT) was used for reconstruction after scans. In the vertebra, the trabecular bone was analyzed between 235 and 229 μm from the lower ends of the pedicles. In the femur, trabecular bone proximal to the distal growth plate was selected for analysis within a conforming volume of interest (cortical bone excluded), starting 426 μm from the growth plate and extending a further longitudinal distance of 135 μm in the proximal direction. Cortical measurements in the femur were performed in the diaphyseal region, beginning at 5.23 mm from the growth plate and extending an additional longitudinal distance of 135 μm in the proximal direction. The data were analyzed using the CTAn software (version 1.13.2.1, Bruker MicroCT). For each time point, age-matched naive littermates served as the reference point, set to 100%, with the values for the irradiated mice expressed relative to this baseline.

### Peripheral quantitative computed tomography (pQCT)

Trabecular and cortical bone in the femur and tibia were analyzed from euthanized mice, both in long-term and acute experiments, using the Stratec pQCT XCT Research M (software version 5.4; Norland, Fort Atkinson, WI, USA) at a resolution of 70 μm. The trabecular bone region was defined in the metaphysis area distal to the growth plate in the tibia and proximal to the growth plate in the femur. This area was designated as 2.6% of the length of the bone from the growth plate, and only the inner area, making up 45% of the total cross-sectional area, was defined as trabecular bone. Cortical thickness was determined by analyzing scans of the mid-diaphyseal region.

### Murine serum analyses

Blood was collected in blood collection tubes containing a silicon serum separator (Microvette 500 Z-Gel, Sarstedt, Nümbrecht, Germany). Serum levels of total cholesterol in mice were measured using commercial fluorometric assay kits (Sigma-Aldrich CS0005). Enzyme-linked immunosorbent assays (ELISAs) were conducted to quantify C-terminal type I collagen fragments (CTX-I), indicative of bone resorption, (Immunodiagnostic Systems, East Boldon, UK), procollagen type I N pro-peptide (PINP), a serum marker for bone formation (Immunodiagnostic Systems), and immune cytokines IL-6 (Invitrogen) and TGF-β1 (Invitrogen).

The Proteome Profiler Mouse XL Cytokine Array Kit (R&D Systems) was used to determine changes in serum protein levels following the manufacturer’s instructions. One pooled control serum from naive mice (*n* = 5) was compared to both pooled sera from mice 2 and 12 weeks after irradiation (*n* = 5). The pooled control serum from naive mice served as the baseline, and the results are reported as the log2 change in relative units compared to serum from naive mice. We quantified and normalized the background signal for each spot using Quick Spots (OptimEyes version 25.5.2.3) and ImageJ (version 1.8.0) software. From the 111 analytes, we selected cytokines, chemokines, and growth factors identified in the literature that are known to play a role in skeletal turnover and inflammation. Serum steroid concentrations were measured using high-sensitivity liquid chromatography-tandem mass spectrometry (LC-MS/MS) as described previously [32].

### Histological examination

Formaldehyde-fixed bone tissue was decalcified in 10% EDTA for 3 weeks. The tibia was then embedded in paraffin and sectioned into 4-μm-thick sections. Hematoxylin and eosin (HE) staining was performed. Adipocytes were identified as empty white round holes, and osteoblasts as cuboidal cells on the bone surface in the HE staining. TRAP staining was conducted to detect osteoclasts. In brief, the slides were pre-warmed at 60 °C for 30 min and then allowed to cool to room temperature. The slides were subsequently washed in xylene, followed by ethanol, and finally with water. Sections were incubated in 0.2 M acetate buffer (pH 5), followed by 1.5 h in TRAP buffer, a solution of naphthol, red-violet, 0.1 M acetate buffer, 0.3 M sodium tartrate, and Triton X-100, and finally counterstained with fast green and then mounted using Fluoromount Antifade (Sigma-Aldrich). Osteoclasts were defined as TRAP-positive cells on the surface of the bone tissue. The TUNEL assay (In Situ Cell Death Detection Kit Fluorescein, Roche Diagnostics) was performed according to the manufacturer’s instructions. Briefly, slides were prepared by incubating at 60 °C for 60 min, followed by rehydration through sequential washes in xylene, 99% ethanol, 95% ethanol, 70% ethanol, and water. The sections were treated with proteinase K (1:2000 in PBS) at 37 °C for 40 min in a shaking water bath, followed by three 10-min washes in PBS. Labeling was accomplished using a prepared enzyme and label solution mix, with tissues incubated at 37 °C for 1.5 h under parafilm and protected from light. Final washing was performed in PBS (3 × 10 min).

Images were taken in the proximal epiphyseal and metaphyseal areas of the tibia using a Zeiss LSM780 confocal laser scanning microscope with a Zeiss C-Apochromat × 40/1.20 objective (Carl Zeiss). Post-acquisition image processing was performed blinded to the groups using Zeiss Zen 3.1 (blue edition) software, with a focus on the total epiphyseal bone and a fixed region of interest measuring 225 μm², located 400 μm from the growth plate in the middle region of the bone.

### Gene expression

RNA extracted from cortical bone (tibia) and bone marrow was obtained using Trizol Reagent (Sigma, St. Louis, MO, USA), followed by the RNeasy Mini QIAcube Kit (Qiagen, Hilden, Germany). This RNA was reverse-transcribed into cDNA utilizing the Applied Biosystems High-Capacity cDNA Reverse Transcription Kit (Applied Biosystems, Waltham, MA, USA). Quantitative PCR (qPCR) was performed using the StepOnePlus Real-Time PCR system (Applied Biosystems). Predesigned probes for *Bax* (Mm00432051_m1), *Tgf-β1* (Mm01178820_m1), *Rank* (Mm00446427_m1), *Rankl* (Mm00441908_m1), and *Opg* (Mm00435452_m1) from Applied Biosystems were utilized. The mRNA abundance of each gene was calculated using the ΔΔCt method and normalized to the expression of *18S* ribosomal RNA (4310893E) (Applied Biosystems).

### Cell preparation and flow cytometry

To prepare cells for flow cytometry analysis, bone marrow cells were flushed out and harvested from the femoral bone using a 25 G syringe with PBS. Splenocytes were isolated, and a single-cell suspension in PBS was obtained by passing them through a 70-µm cell strainer. The pelleted cells were resuspended in a 0.1 M Tris buffer (pH 7.4) solution to lyse erythrocytes and washed in PBS. The total number of leukocytes was determined using a cell counter (Sysmex, Europe GmBH, Norderstedt, Germany). The fluorochrome-conjugated anti-mouse antibodies used for flow cytometry analysis included: allophycocyanin (APC)-conjugated F4/80, APC-Cy7-conjugated anti-CD8, phycoerythrin (PE)-conjugated anti-CD19 (eBioscience, Thermo Fisher Scientific, Gothenburg, Sweden); APC-cyaninine5 (Cy5)-conjugated anti-B220, Brilliant Violet 421-conjugated anti-CD69, V450-conjugated anti-CD11b (Becton Dickinson and Company, Franklin, NJ, USA); and PE-Cy7-conjugated anti-B220, APC-conjugated anti-CD4 and PE-Cy7-conjugated anti-CD8, PerCP-conjugated anti-Gr-1 (eBioscience, Thermo Fisher Scientific, Gothenburg, Sweden), FITC-conjugated Annexin V (Becton Dickinson and Company, Franklin, NJ, USA) and 7AAD (Becton Dickinson and Company, Franklin, NJ, USA). Annexin V and 7AAD staining was performed in a 1x Annexin V Binding Buffer containing 0.1 M Hepes (pH 7.4), 1.4 M NaCl, and 25 mM CaCl_2_ (BD Biosciences). Flow cytometry analyses were performed using FACSVerse (Becton Dickinson) and FlowJo (version 10.6.2) (detailed gating strategies in Fig. S5).

### Apoptosis induction

Thymocytes were isolated as apoptotic targets from 4-week-old C57BL/6 female mice, as described before [33]. Cells cultured at a density of 10^7^ cells/mL in α-MEM medium supplemented with 2 mM glutamine, 100 U/mL penicillin, and 100 µg/mL streptomycin. Apoptosis was induced by incubating thymocytes in a serum-free medium for 24 h. Freshly isolated thymocytes were used as viable controls for the flow cytometry measurements. The levels of apoptotic and necrotic cells were assessed using 7-AAD/Annexin V staining, and flow cytometry analyses were performed with a FACSVerse flow cytometer (Becton Dickinson) and analyzed using FlowJo software (version 10.6.2) (see detailed gating strategies in Fig. S6A).

### Osteoclast differentiation from bone marrow-derived macrophages (BMMs)

Murine osteoclast differentiation was studied in vitro using RANKL-induced differentiation. Bone marrow-derived macrophages (BMMs) were obtained from the bone marrow of C57BL/6 mice, aged between 8–12 weeks, and cultured in suspension dishes (Corning Inc.) with complete α-MEM medium (Gibco; 12561056), supplemented with 10% heat-inactivated fetal bovine serum (FBS, Sigma-Aldrich; F7524), 100 U/mL penicillin, 100 µg/mL streptomycin (Gibco), and 2 mM GlutaMAX (Gibco). Additionally, the medium included 30 ng/mL macrophage colony-stimulating factor (M-CSF) (R&D Systems). After 2 days, nonadherent cells were washed away with PBS, and adherent BMMs were detached from the dish using 0.02% EDTA in PBS (Sigma-Aldrich). BMMs were then spot-seeded in 96-well plates at a density of 5000 cells per 5 µL in the center of the wells and incubated with 200 µL of media. All cultivations were stimulated with 30 ng/mL M-CSF for survival, 4 ng/mL RANKL (R&D Systems; 462-TEC-010) to induce osteoclast differentiation, and additionally stimulated with 0.5 ng/ml TGF-β1 (R&D Systems; 7754-BGICF), 5 μM TGF-β1 inhibitor (Sigma-Aldrich SB431542) or apoptotic cells from thymocytes, at a 1:5 monocytes: apoptotic thymocytes.

The medium was changed after 3 days, when some of the cells differentiated into multinucleated preosteoclasts or osteoclasts. An additional 24–36 h with different stimuli was applied before the cultivation was stopped.

### Osteoclast differentiation from RAW cells

RAW 264.7 cells (ATCC; TIB-71) were cultured in complete Dulbecco’s Modified Eagle’s Medium (DMEM, Gibco), supplemented with 10% heat-inactivated fetal bovine serum (FBS, Sigma-Aldrich; F7524), 100 U/mL penicillin, and 100 µg/mL streptomycin (Gibco; 15140122). To induce osteoclast differentiation, RAW 264.7 cells were re-suspended in α-MEM (Gibco), spot-seeded into 96-well plates at a density of 8000 cells/5 µL in the center of each well, and treated with 2 ng/mL RANKL (R&D Systems). The cultivation process lasted a total of 3 days, with media changes on day 1.

### Osteoclast analysis

After 3 days of cultivation, TGF-β1 protein expression (Invitrogen 88-50690-22) and TRAP5b activity (Bone TRAP, Immunodiagnostic Systems) were measured in the culture supernatant from BMM differentiated osteoclasts. To study gene expression, RNA was extracted from cells lysed in RLT buffer using the RNeasy Micro QIAcube Kit (Qiagen, Hilden, Germany). The RNA was transcribed into cDNA as described above, and qPCR was performed using the StepOnePlus Real-Time PCR system (Applied Biosystems). Predesigned probes for *Nfatc1* (Mm00479445_m1), *Acp5* (Mm0047568_m1), and *Rank* (Mm00446427_m1), from Applied Biosystems were utilized. The mRNA abundance of each gene was calculated using the ΔΔCt method and normalized to the expression of *Eef2* ribosomal RNA (Mm01171435_gH) (Applied Biosystems) and expressed relative to the mean expression in the M-CSF-treated group.

The formation of osteoclasts was evaluated by TRAP staining (Sigma-Aldrich) after 4–5 days of cultivation of BMM and RAW cells. Pictures were taken with a Jenoptik Gryphax Camera in a Nikon Eclipse 80i microscope, and osteoclasts were identified as TRAP-positive cells with three or more nuclei. The number, area, and perimeter of osteoclasts were analyzed using the Zeiss Zen 3.1 (blue edition) software and presented as % of the mean of the M-CSF and RANKL-stimulated sample.

### Phagocytosis assay

BMM-derived cells were prepared as previously described. The cells were spot-seeded at a density of 5000 cells/5 μL, and after cell attachment, the media volume was adjusted to a total of 200 μL in each well of an 8-well culture slide (Falcon Culture Slides, 354108). The cells were then incubated with 30 ng/mL M-CSF, either alone or in combination with 4 ng/mL RANKL. Thymocytes were labeled with 5 μM Vibrant CFDA (Molecular Probes) for 30 min at room temperature before being added to the BMM cultures at a 1:5 cell ratio. Phagocytosis was allowed to proceed for either 60 min (short-term assay) or throughout a 3-day differentiation period (long-term assay) at 37 °C in a 5% CO₂ atmosphere. For the short-term (60-min) assay, cells were stained with CellMask Orange (Invitrogen) at a final concentration of 2 μg/mL for 15 min, and nuclei were counterstained with Hoechst 33342 (Molecular Probes-Invitrogen) at a final concentration of 5 μg/mL, following the manufacturer’s instructions. After the co-culture period, non-internalized thymocytes were thoroughly washed away with PBS, and the cells were fixed with 4% paraformaldehyde (PFA) for 10 min at room temperature. Finally, slides were mounted using ProLong Antifade Mountant (Molecular Probes) for imaging. Cells containing two nuclei were classified as pre-osteoclasts, while those containing three or more nuclei were classified as osteoclasts.

### Statistics

All statistical analyses were conducted using GraphPad Prism software (GraphPad Software Inc., La Jolla, CA, USA). All except the sex steroids, where a limited sample size was used, were normally distributed. Power analysis using P*Power 3.1 showed that at least six mice per group are needed to achieve 80% power to detect a 1.5-standard deviation (SD) change between irradiated and HSCT mice compared to naive in computer tomography investigation, based on preliminary studies. Variance is similar in irradiation and HSCT groups as in naive controls. Sample sizes are provided in the figure and table legends, and each sample is indicated in each figure.

Statistical differences in sex steroids were checked using the Mann–Whitney *U* test. A two-sided unpaired Student’s *t* test was used to determine statistical differences between two independent groups (naive and irradiated). For multigroup comparisons, one-way ANOVA was performed, followed by Dunn’s test with Šidák correction to compare the control group against each stimulation group (naive versus the different time points post-irradiation). ANOVA followed by Tukey’s comparison was used for the cultivated cells, comparing all groups to each other. Figures showing data mean ± standard deviation (SD), and significance is indicated as follows: *p < 0.05, **p < 0.01, and ***p < 0.001.

## Results

### Total body irradiation has a long-term negative impact on bone mass and uterus weight in female C57BL/6 mice

In this study, we examined the effects of total body irradiation (TBI) followed by hematopoietic stem cell transplantation (HSCT) on female C57BL/6 mice compared to age-matched sibling littermate naive control mice (Fig. 1A). One week after irradiation, both the irradiated and HSCT mice experienced temporary fluctuations in body weight, after which their weight returned to that of the naive mice (Fig. 1B and Table 1).

**Table 1:** Body and organ weight.

		Post-irradiation time			
		1 d	2w	6w	12w
Body weight (g)	Naive	20.64 ± 1.56	20.11 ± 2.00	21.87 ± 1.92	22.31 ± 2.07
	Irradiated	20.13 ± 1.61	18.77 ± 2.18	20.93 ± 0.98	21.37 ± 1.26
Spleen /body weight (mg/g)	Naive	3.05 ± 0.39	3.45 ± 0.41	3.23 ± 0.34	3.58 ± 0.40
	Irradiated	1.67 ± 0.21***	4.01 ± 0.42*	3.97 ± 0.51**	3.50 ± 0.31
Thymus/body weight (mg/g)	Naive	1.77 ± 0.41	2.44 ± 0.30	2.02 ± 0.41	1.76 ± 0.37
	Irradiated	0.76 ± 0.18***	1.94 ± 0.41**	2.43 ± 0.47*	1.61 ± 0.37
Uterus/body weight (mg/g)	Naive	2.90 ± 1.30	4.27 ± 1.87	2.84 ± 1.14	2.21 ± 0.58
	Irradiated	2.48 ± 0.64	2.59 ± 0.66*	0.83 ± 0.28***	0.77 ± 0.21***

Sample sizes ranged from *n* = 8 to 12. Data are presented as mean ± SD. Statistical analysis was performed using the Student’s *t* test to assess differences between the irradiated and naive mice at each time point. Significance levels are indicated as *p < 0.05; **p < 0.01; ***p < 0.001.

Total and lumbar spine areal-bone mineral density (BMD) was measured in the same mice over time, revealing a significant decrease from 4 weeks post-irradiation and HSCT compared to naive controls (Fig. 1C).

A significant reduction in spleen and thymus weight was observed within a day after irradiation exposure (Table 1). Two and 6 weeks post-irradiation and HSCT, there was a notable increase in spleen weight. In contrast, thymus weight was reduced 2 weeks after irradiation but returned to levels similar to those of naive mice 6 weeks after irradiation and HSCT. Meanwhile, although uterine weight remained unchanged the day after irradiation, significant atrophy was noted starting 2 weeks after irradiation and HSCT and continued through the 12-week investigation. No differences were observed in gonadal fat and liver weight.

Micro-computed tomography (µCT) conducted 1 day post-irradiation indicated no direct adverse effects in relative changes on femoral trabecular bone volume per tissue volume (Tb. BV/TV) (Fig. 1D, F). By the second week post-irradiation and HSCT, changes in bone density were observed, including a relative reduction in femur Tb. BV/TV, decreased trabecular number, increased trabecular separation, and decreased thickness (Fig. 1D–F). These alterations in relative changes persisted 6 weeks post-irradiation, except for trabecular thickness. By the 12-week mark, the Tb. BV/TV returned to normal compared to controls, but changes in trabecular number and separation remained. Absolute change in µCT and relative changes in pQCT analysis confirmed the µCT findings, showing a decrease in both tibial and femoral trabecular BMD that began 2 weeks post-irradiation HSCT and persisted throughout the 12-week period (Fig. S1A–D). Furthermore, µCT assessment observed a reduction in trabecular bone in the axial vertebrae 2 weeks post-irradiation and HSCT (Fig. S2).

Furthermore, the cortical thickness in the femur significantly decreased by 2 weeks post-irradiation and remained reduced until 12 weeks, as determined by μCT (Fig. 1G). The same pattern was observed in the tibia and femur through pQCT analysis, but there was no significant alteration 2 weeks post-irradiation (Fig. S1A, B).

Mice exhibited a significant change in bone remodeling markers in serum. The bone resorption marker CTX-I increased at 2- and 6-weeks post-irradiation and HSCT (Fig. 1H). The bone formation marker PINP decreased 2 weeks after irradiation, but an unexpected induction was observed at 6 weeks (Fig. 1H).

### Total body irradiation alters bone cell populations and promotes adipogenesis

**Fig. 2:**
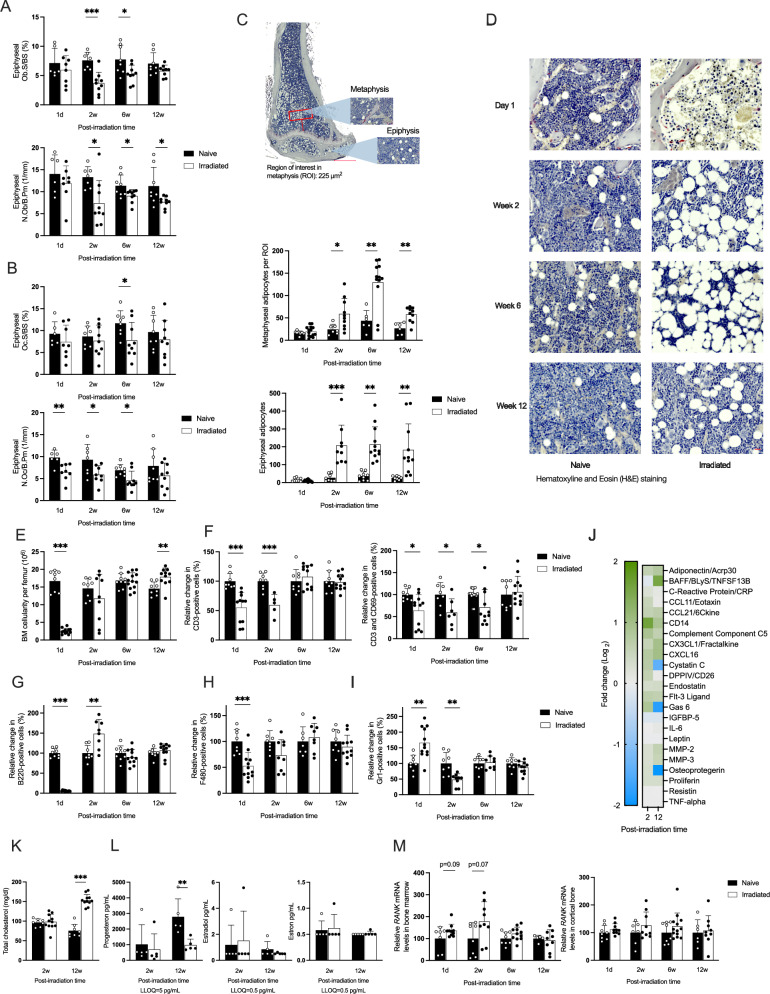
Longitudinal analysis of bone cells, bone marrow composition and serum proteins, following irradiation and hematopoietic stem cell transplantation (HSCT). **A** Osteoblast surface per bone surface (Ob S/BS) and number of osteoblasts per bone perimeter (N Ob/B Pm) in the total proximal epiphyseal part of the tibia. **B** Osteoclast surface per bone surface (Oc S/BS) and number of osteoclasts per bone perimeter (N Oc/B Pm) in the proximal total epiphysis of the tibia. **C** Quantification of bone marrow adipocyte number in a region of interest (ROI) in the metaphysis and the total proximal epiphysis of the tibia. **D** Hematoxylin and eosin (H&E) staining of the tibia. **E** Number of bone marrow cells per femur. **F** Relative change in frequency of CD3^+^ T cells and CD69^+^ activated T cells gated on the CD19^−^ cell population. **G** Relative change in frequency of B220^+^ B cells gated on the CD3^−^ cell population. **H** Relative change in frequency of F480^+^ monocytic subset gated from the CD3^−^CD11b^+^ population. **I** Relative change in frequency of Gr1^+^ neutrophil cells gated from the CD3^−^CD11b^+^ population. **J** Protein composition in serum post-irradiation and HSCT was detected by Mouse Cytokine microarray membrane analysis. Log_2_ fold change was calculated using the corrected pixel density. **K** Comparative analysis of total serum cholesterol levels. **L** Measurement of female sex steroids progesterone, estradiol, and estrone levels in the serum in a subset of the mice (*n* = 5). **M** Analysis of the relative gene expression of *Rank* in bone marrow and cortical bone. Statistical analysis was performed using Student’s *t* test to assess differences at each time point except for sex steroids, where Mann–Whitney was performed due to not normally distributed values. The number of samples ranged from *n* = 8–12. Data are presented as mean ± SD. Significance levels are indicated as *p < 0.05, **p < 0.01, ***p < 0.001.

Following the observed changes in bone density and serum markers of bone remodeling, we examined osteoclasts and osteoblasts in the tibial epiphyseal bone (Fig. 2A, B). One day after irradiation and HSCT, there were no immediate changes in the osteoblasts (Fig. 2A). Two weeks after irradiation and HSCT, we observed a reduction in the osteoblast surface area per bone surface and the number of osteoblast surface area per bone surface and the number of osteoblasts per bone perimeter, which persisted throughout the observation period. The number of osteoclasts per bone perimeter declined immediately after irradiation and remained low over 6 weeks post-irradiation, while the osteoclast surface per bone surface showed significant differences only at 6 weeks post-irradiation (Fig. 2B). Adipocytic expansion within the bone marrow in both metaphyseal and epiphyseal bone occurred at 2 weeks and persisted throughout the investigation (Fig. 2C, D).

A distinct reduction of bone marrow cells was observed in the histological sections 1 day after irradiation (Fig. 2D, top panels). This reduction was confirmed by counting the cellularity in the femur (Fig. 2E). The cellularity was restored at 2 weeks and exceeded the naive mice cellularity 12 weeks post-irradiation and HSCT (Fig. 2E). Not only did the absolute numbers decrease, but the frequency of several bone marrow cell types, such as CD3^+ ^T cells, activated CD69^+^ T cells, B220^+^ B cells, and the F480^+^ macrophage subset, also declined 1 day after irradiation (Fig. 2F–H). Conversely, the frequency—but not the numbers—of Gr1+ neutrophils rapidly increased 1 day after irradiation but decreased 2 weeks post-irradiation and HSCT (Fig. 2I). The frequency of CD3^+^ T cells and F480^+^ monocytes remained lower than in the naive mice 2 weeks post-irradiation and HSCT. However, the Frequency of B220^+^ B cells increased 2 weeks post-irradiation and HSCT compared to the naïve mice. By 12 weeks post-irradiation and HSCT, the frequencies of all investigated cell types had returned to normal levels. This notable restoration of cell frequencies over time was also observed in the spleen, where a similar rapid decrease in lymphocytes and elevated levels of B cells were present at 2 weeks post-irradiation (Fig. S3A–D). The elevated Gr1^+^ neutrophil population was also observed in the spleen, which returned to similar levels as naive mice 6 weeks post-irradiation and HSCT (Fig. S3A–D).

To analyze the differential protein expression changes affecting systemic bone turnover and immune regulation, we performed a semi-quantitative microarray on serum samples, comparing 2- and 12-week post-irradiation and HSCT. The serum levels of B-cell activating factor (BAFF) were elevated, while cystatin C, growth arrest-specific protein 6 (GAS6), and osteoprotegerin (OPG) levels decreased at 12 weeks compared to naive mice (Fig. 2J and Table S1). Interestingly, key inflammation markers, including IL-6, remained unaffected, as confirmed by ELISA (Figs. 2J and S3E). Total serum cholesterol levels increased 12 weeks after irradiation (Fig. 2K). Serum progesterone levels decreased 12 weeks post-irradiation and HSCT, but estradiol levels remained unchanged and were undetectable in several mice from both naive and irradiated groups (Fig. 2L). Finally, the gene expression of *Rankl* and *Opg*, as well as the *Rankl/Opg* ratio, was not affected by irradiation (Fig. S3G). *Rank* expressions in bone marrow was borderline elevated 1 day and 2 weeks post-irradiation, but no change was observed in cortical bone (Fig. 2M).

### Persistent TUNEL-positive cells were detected in the bone post-irradiation

**Fig. 3:**
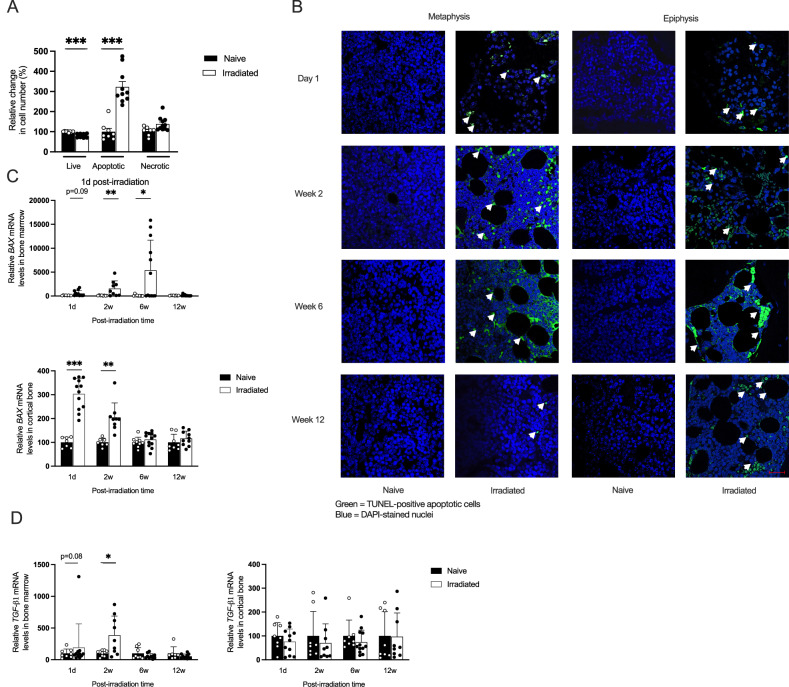
Characterization of irradiation-induced cell death in bone and bone marrow. **A** Flow cytometric detection of relative changes in the number of apoptotic and necrotic cells in bone marrow 1 day post-radiation, utilizing Annexin V-FITC/7-AAD staining. Apoptotic cells were identified as Annexin V^+^ (Annexin V^+^), necrotic cells as both Annexin V and 7^-^AAD^+^ (Annexin V^+^7AAD^+^), live cells as negative for both Annexin V and 7^-^AAD (Annexin V^−^7AAD^−^), and cell fragments or debris were characterized by high 7-AAD density. **B** Apoptotic cells visualized by TUNEL staining in the distal femur metaphysis and epiphysis. All images are captured at ×40x magnification with a scale bar of 40 µm. **C** Analysis of the relative gene expression of *Bax* gene (key protein in initiating apoptosis) in bone marrow and cortical bone. **D** Analysis of the relative gene expression level of *TGF-β1* gene (multifunctional pro-apoptotic cytokine) in bone marrow and cortical bone. The number of samples ranged from *n* = 8–12. Data are presented as mean ± SD. Statistical significance was determined using Student’s *t* test at each time point, with *p* values indicated as *p < 0.05, **p < 0.01, ***p < 0.001.

As expected, the cellularity of the bone marrow dramatically decreased 1 day after irradiation (Fig. 2D, E). Flow cytometry analysis revealed an increase in apoptotic cells at this time point, but no change in necrotic cells (Fig. 3A). TUNEL staining confirmed the presence of apoptotic cells in the epiphysis and metaphysis of the bone marrow, as well as within the bone tissue of the tibia, 1 day after irradiation, and surprisingly, this presence persisted over the 12-week investigation (Fig. 3B). The number of TUNEL-positive cells was elevated at 2- and 6-weeks post-irradiation and HSCT and was also present 12 weeks later. The apoptotic cells in the bone marrow were accompanied by an upregulation of pro-apoptotic *Bax* gene expression in both bone marrow and cortical bone 1 day post-irradiation, remaining elevated for 6 weeks in the bone marrow and for 2 weeks in cortical bone (Fig. 3C). In addition, *Tgf-β1* gene expression was elevated in the bone marrow post-irradiation, but not in cortical bone (Fig. 3D). Furthermore, TGF-β1 levels in serum were borderline elevated 1 day after irradiation (Fig. S3F).

### Total body irradiation showed no immediate effect on bone microarchitecture but altered the bone marrow cells

**Fig. 4:**
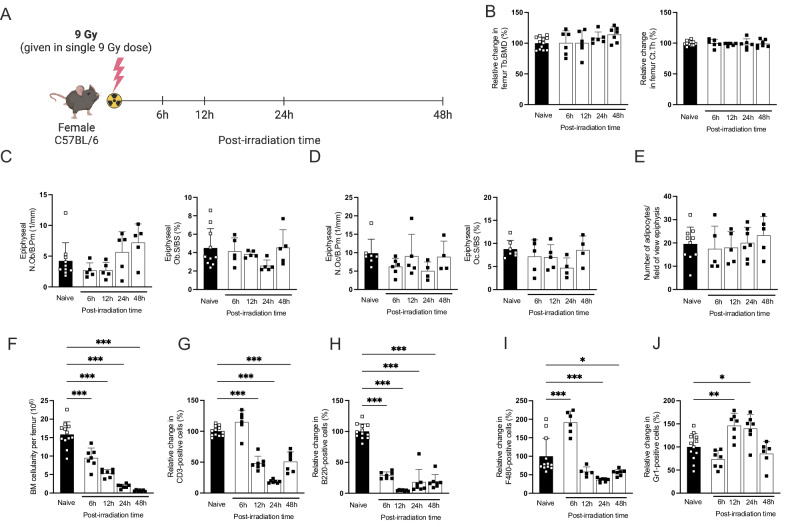
Acute effects of irradiation on bone parameters, bone cells, and bone marrow cells. **A** Experimental design of the acute radiation scheme performed on 9-week-old mice. The mice were euthanized at 6, 12, 24, and 48 h (48 h) post-irradiation. **B** Peripheral Quantitative Computed Tomography (pQCT) measurements of trabecular Bone Mineral Density (Tb.BMD) and cortical thickness (Ct.Th) in irradiated and naive mice. **C** Number of osteoblasts per bone perimeter (N Ob/B Pm) and osteoblast surface per bone surface (Ob S/BS) in the distal epiphyseal part of the tibia. **D** Number of osteoclasts per bone perimeter (N Oc/B Pm) and osteoclast surface per bone surface (Oc S/BS) in the total proximal epiphysis of the tibia. **E** Quantification of bone marrow adipocyte number in the total proximal epiphysis of the tibia. **F** Number of bone marrow (BM) cells per femur. **G** Relative change in CD3^+^T cells gated on the CD19− cell population. **H** Relative change in B220^+^ B cells gated on the CD3^−^ cell population. **I** Relative change in F480^+^ monocytic subset gated from the CD3^−^CD11^+^ population. **J** Relative change in Gr1^+^ neutrophil cells gated from the CD3^−^CD11^+^ population. For statistical analyses, one-way ANOVA and Dunnett’s multiple comparisons test were used to compare the mean values of a naive group with each time point. The number of samples ranged from *n* = 8 to 12. Data are presented as mean ± SD, and significance is indicated as *p < 0.05, **p < 0.01, ***p < 0.001.

To further investigate the immediate changes after irradiation that may develop into long-term effects, we conducted an experiment using a single irradiation of 9 Gy to elucidate the underlying mechanisms (Fig. 4A). In response to irradiation, body weight remained stable during the first 48 h (Table 2). Femur trabecular bone mineral density and cortical thickness determined by pQCT were unaffected within this limited timeframe (Fig. 4B).

**Table 2:** Body and organ weight.

		Post-irradiation time			
	Naive	6 h	12 h	24 h	48 h
Body weight (g)	20.11 ± 0.98	18.99 ± 0.80	19.89 ± 0.84	18.99 ± 1.67	19.20 ± 1.17
Spleen/body weight (mg/g)	3.13 ± 0.37	2.49 ± 0.38***	2.04 ± 0.35***	1.59 ± 0.15***	1.41 ± 0.25***
Thymus/body weight (mg/g)	2.35 ± 0.55	2.00 ± 0.22	1.65 ± 0.15***	0.87 ± 0.17***	0.42 ± 0.11***
Uterus/body weight (mg/g)	2.93 ± 1.30	3.06 ± 1.28	3.24 ± 0.83	2.85 ± 2.05	1.62 ± 0.10
Liver/body weight (mg/g)	42.24 ± 3.46	39.77 ± 3.95	37.15 ± 2.05**	43.32 ± 3.48	42.83 ± 2.27

Sample sizes ranged from *n* = 8 to 12. Data are presented as mean ± SD. Statistical analysis was performed using the Student’s *t* test to assess differences between the irradiated and naive mice at each time point. Significance levels are indicated as **p < 0.01; ***p < 0.001.

A reduction in spleen weight was noted in the irradiated group as early as 6 h post-irradiation, with the decrease continuing up to 48 h (Table 2). Thymus weight was reduced 12 h post-irradiation, with continuing decreases up to 48 h. Significant alterations in liver weight were observed only 12 h after irradiation. Uterine weight remained unaffected. No changes were observed in the numbers of osteoblasts, osteoclasts, and adipocytes, nor the size of osteoclasts and osteoblasts in the tibial epiphyseal region (Fig. 4C–E). Bone marrow cellularity gradually decreased in the irradiated group compared to the naive (Fig. 4F). The frequency of the CD3^+^ T cell population decreased 12 h post-irradiation (Fig. 4G), while the B220+ B cell population showed greater radiosensitivity as its frequency dramatically decreased immediately after irradiation (Fig. 4H). For the F480^+^ monocytic population, a rapid increase in cell frequency was observed 6 h after irradiation, followed by a decrease at 24- and 48-h post-irradiation (Fig. 4J). Finally, the Gr1^+^ neutrophil population increased at 12- and 24-h post-irradiation (Fig. 4K). This same pattern of alteration in cell frequencies was also observed in the spleen, with a similar rapid decrease in lymphocytes and an increase in neutrophils and monocytes (Fig. S4).

**Fig. 5:**
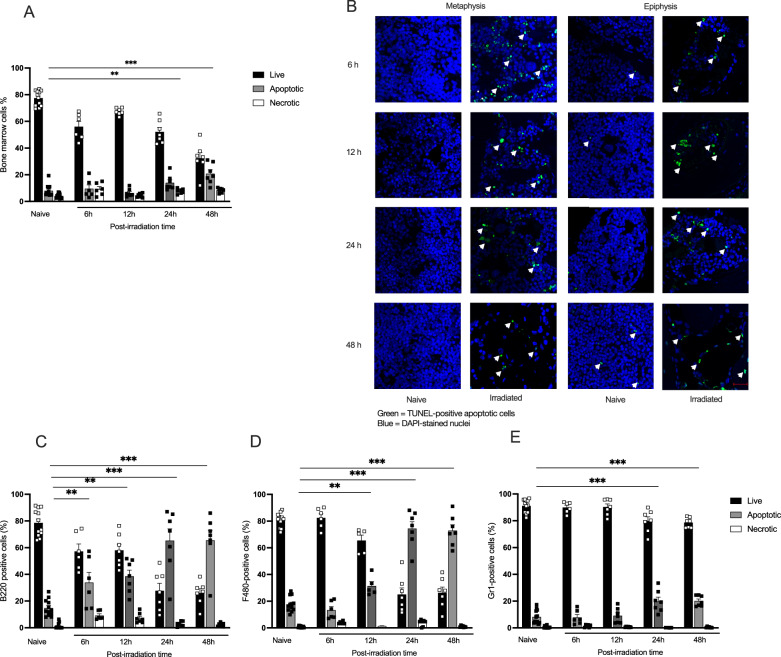
Acute effects of irradiation on apoptosis and necrosis in bone marrow. **A** Flow cytometric detection of relative change in apoptosis and necrosis in bone marrow cells post-radiation, utilizing Annexin V-FITC/7-AAD staining. Apoptotic cells were identified as Annexin V+ (Annexin V+), necrotic cells as both Annexin V and 7AAD^+^ (Annexin V^+^7AAD^+^), live cells as negative for both Annexin V and 7-AAD (Annexin V^–^7AAD^–^), and cell fragments or debris were characterized by high 7-AAD density. **B** Representative images of TUNEL staining of the tibia in the metaphysis and epiphysis areas. **C** Percentage of live, apoptotic, and necrotic cells in B220^+^ B cells gated on the CD3^−^ cell population. **D** Percentage of live, apoptotic, and necrotic cells in F480+ monocytic subset and **E** Gr1^+^ neutrophil cells gated from the CD3^−^CD11^+^ population. Statistical analysis involved a one-way ANOVA followed by the Dunn–Šidák post hoc test. Data are presented as mean ± SD, and significance is indicated as **p < 0.01, ***p < 0.001 compared to the naive mice live population.

Flow cytometry analysis showed that most cells in the bone marrow of naive mice were alive (Fig. 5A). After irradiation, the frequency of viable cells decreased in line with the reduced number of bone marrow cells, while apoptotic cells increased up to 48 h post-irradiation. TUNEL staining revealed dying cells in the bone tissue of both the epiphyseal and metaphyseal regions as early as 6 h post-irradiation, persisting across all subsequent time points (Fig. 5B).

Within 6 h post-irradiation, B cells showed a sharp increase in apoptosis, peaking at 24 h (Fig. 5C). Monocytes showed a significant rise in apoptosis at 12 h (Fig. 5D). Neutrophils displayed a limited but significant increase in apoptotic response at 24- and 48-h post-irradiation (Fig. 5E).

### Pre-osteoclasts can engulf apoptotic cells during in vitro differentiation

Apoptotic cells exists in the bone marrow compartment during the 12-week follow-up after irradiation may influence bone resorption. To mimic this effect, apoptotic lymphocytes from the thymus were added to osteoclast cultures in vitro. Primary murine bone marrow macrophage (BMM) and RAW264.7 cells were differentiated with RANKL to induce osteoclast differentiation, or they were maintained as macrophages as a control.

**Fig. 6:**
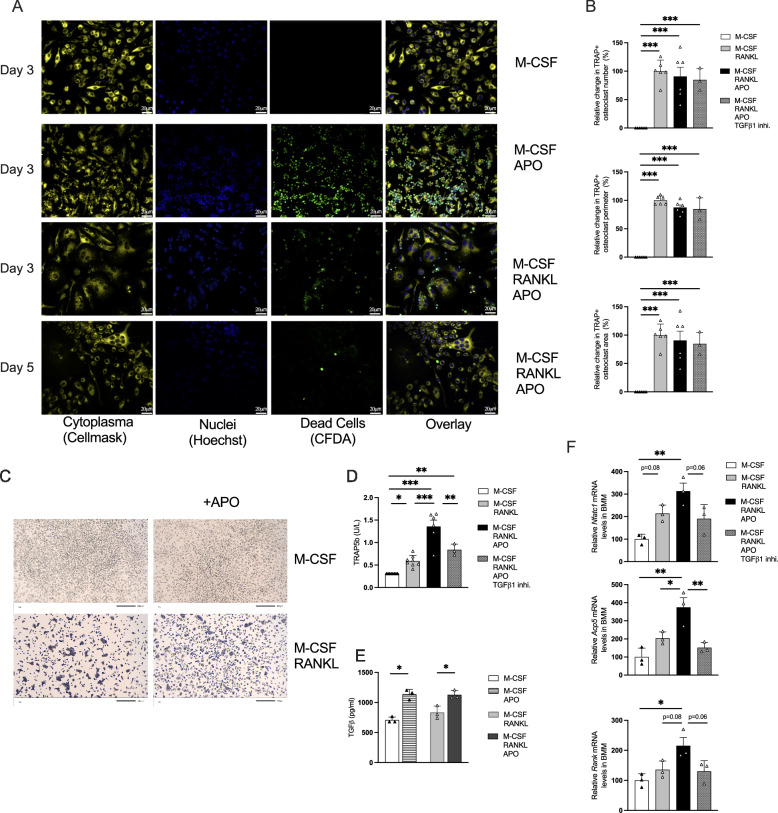
Apoptotic cell engulfment enhances osteoclastogenic gene expression in bone marrow–derived macrophages. **A** Representative fluorescence images of the osteoclast phagocytosis assay in bone marrow–derived macrophages. BMM were stimulated with macrophage colony-stimulating factor (M-CSF), apoptotic thymocytes (APO), and receptor activator of nuclear factor kappa-B ligand (RANKL). Apoptotic thymocytes were labeled with CFDA (green), cytoplasm with CellMask Orange (orange), and nuclei with Hoechst 33342 (blue). Separate channels and merged overlays are shown. Cells with two nuclei were classified as pre-osteoclasts, and cells with three or more nuclei as osteoclasts. **B** Relative changes in the number, perimeter, and area of multinucleated TRAP+ cells after differentiation, with or without APO and transforming growth factor beta1 (TGF-β1) inhibitor. **C** Representative images of tartrate-resistant acid phosphatase (TRAP)-stained osteoclast cultures. **D** TRAP5b and **E** TGF-β protein expression in the supernatant of osteoclast cultures. **F** Analysis of relative gene expression of *Nfatc1* (major transcription factor in osteoclastogenesis)*, Acp5* (TRAP critical for osteoclastogenesis), and *Rank* (a receptor specific to osteoclasts and progenitors) gene. Statistical analysis involved a one-way ANOVA followed by Tukey’s multiple post hoc test. Data are presented as mean ± SD, and significance is indicated as *p < 0.05, **p < 0.01, ***p < 0.001 (*n* = 3–8).

When apoptotic cells were added to the BMM culture at the start of the cultivation, they were evenly distributed throughout the well (Fig. S6B). After 3 days, BMM macrophages cocultured with apoptotic cells displayed significant engulfment. A higher number of apoptotic cells remained along the border of the well compared to the center, where the cells were spot-seeded. When RANKL was added to promote osteoclast differentiation, the engulfment of apoptotic cells was observed, albeit to a lesser extent than in control macrophages. The direct engulfment process was further examined when apoptotic cells were added just 60 min after 3 days of BMM differentiation. During this time, phagocytosis occurred, with apoptotic cells found inside the macrophage cytoplasm (Fig. 6A). In cultures treated with RANKL for 3 days to promote osteoclast differentiation, both pre-osteoclasts and osteoclasts engulfed apoptotic cells. Interestingly, fully mature osteoclasts with more than three nuclei showed a reduced ability to engulf apoptotic cells 3 and 5 days after RANKL treatment (Fig. 6A).

When BMMs and RAW264.7 cells were cultured with apoptotic cells for 3 days and in the presence of M-CSF alone or together with RANKL, no differences were seen in osteoclast number, area, or perimeter (Figs. 6B, C and S6C, D). However, in BMM cultures exposed to apoptotic cells, TRAP5b levels were elevated in the supernatant after 3 days of differentiation (Fig. 6D). This increase induced by apoptotic cell was accompanied by higher levels of TGF-β1 in the same supernatants, in both M-CSF-treated macrophages and RANKL-stimulated osteoclast cultures (Fig. 6E). Importantly, *Tgf-β1* expression was also higher in bone marrow in vivo, both 1 day and 2 weeks after irradiation (Fig. 3D).

To determine whether the effects induced by apoptotic cells were mediated through TGF-β1, we tested the response of BMM cultures to TGF-β1 stimulation. Although TGF-β1 did not significantly increase the number of osteoclasts and only borderline increased TRAP5b secretion compared to RANKL-stimulated (Fig. S7A, B). The gene expressions of *Acp5* and *Nfatc1* were significantly induced in the BMM cultures, along with *Rank* (Fig. S7C). Importantly, these effects were significantly reduced by the addition of a TGF-β1 inhibitor (Fig. S7A–C).

Apoptotic cell treatment elicited a gene expression pattern similar to *Tgf*-β1 stimulation, with increased levels of *Acp5*, *Nfatc1*, and *Rank* in BMM cultures (Fig. 6F). These responses were counteracted by TGF-β1 inhibition, as evidenced by lower TRAP5b levels, and decreased expression of targeted genes (Fig. 6B, D, F). Overall, these findings indicate that apoptotic cells promote osteoclast activation through a mechanism involving TGF-β1 signaling.

## Discussion

Although extensive research has established that total body irradiation (TBI) disrupts bone homeostasis, the aspects of dead or dying cells have not been studied, and much of the existing literature has focused on either the acute or short-term effects. Studies have shown that irradiation-induced bone loss could be mediated by the dysfunction of bone mesenchymal stem cells [2], increased senescence in both mesenchymal and hematopoietic cells [34], or an increase in the hypoxic bone microenvironment [35]. While these processes are likely central to irradiation-induced bone loss, we also believe that the induction of apoptotic cells may be critical in influencing osteoclasts and bone health. Bone loss is a recognized complication occurring within 8–12 months following hematopoietic stem cell transplantation (HSCT), where massive cell death can be induced by radiation or, now more commonly, chemotherapy [36]. In addition, our collaborators have shown a markedly increased risk of fractures in a large population-based study from Sweden [8]. Our study therefore explored whether the irradiation effect on bone mineral density and bone marrow in female C57BL/6 mice was driven by apoptotic cells during both the acute phase, and up to 12 weeks after irradiation and HSCT. By examining these outcomes in both the acute and extended phases post-irradiation and the impact of apoptotic cells on osteoclastogenesis, our study provides insights into the persistent consequences of irradiation and HSCT on bone homeostasis.

Immune cells and bone cells are closely interconnected, primarily through the RANKL-RANK-OPG axis that regulates osteoclastogenesis. Activated T cells are a major source of RANKL [37], while B cells help maintain balance by producing both RANKL and its decoy receptor OPG [38]. Additionally, immune cells secrete a wide range of cytokines, which influence RANKL expression and shape the local inflammatory microenvironment. We observed a temporary increase in the frequency of neutrophils, indicating acute sterile inflammation that may have briefly affected the marrow microenvironment. Simultaneously, there was a significant decrease in overall bone marrow cellularity and in the frequencies of B cells, T cells, and monocytes, indicating effective depletion. Monocyte levels remained low for 2 weeks after post-irradiation but returned to baseline by 6 weeks. The transient reduction in osteoclast precursors in the bone marrow may explain the lower osteoclast numbers observed despite elevated serum CTX levels during recovery.

The frequency of B cells recovered rapidly and was significantly increased 2 weeks post-irradiation compared to controls. This compensatory response was also evident in elevated serum BAFF levels, increased spleen weight, and enhanced spleen cellularity. Although B cells produce OPG, neither bone marrow expression nor serum protein levels increased 2 weeks post-irradiation, indicating that the observed B cell increase does not affect bone via OPG. Conversely, T cells remained limited, with normalization occurring only 6 weeks post-irradiation. A similar pattern was observed in thymus weight, suggesting a direct link between thymus atrophy and T cell depletion. While T cells are major producers of RANKL, no changes in Rankl expression in the bone marrow were found at any time post-irradiation, nor was there any alteration in the Rankl/Opg ratio in the bone marrow.

Bone marrow cellularity returned to normal 2 weeks post-irradiation and HSCT, then exceeded control group levels by 12 weeks, indicating a compensatory proliferation. However, this increase in absolute immune cell numbers was not accompanied by changes in cytokine expression in the bone marrow, immune cell frequencies, or serum protein levels, suggesting a localized rather than systemic immune response to irradiation.

The uterus showed progressive atrophy 2 weeks post-irradiation, likely linked to the previously observed high radiosensitivity [39, 40]. Decreased serum levels of progesterone further confirmed this uterine atrophy, but estrogen and estradiol levels were undetectable in both naive and irradiated HSCT mice. Cholesterol, a metabolic marker and precursor to sex steroids, was elevated 12 weeks post-irradiation, an interesting observation that requires further investigation.

The high calcium content in bone makes it radiosensitive [3, 4]. In addition, the bone provides a specific environment where hematopoietic and mesenchymal cells regulate each other, and there is evidence that hematopoietic-lineage cells are intricately intertwined with the bone tissue [41]. Irradiation significantly disrupted both trabecular and cortical bone parameters. Total areal bone mineral density declined after 4 weeks, and this decline persisted for up to 12 weeks post-irradiation and HSCT. Cortical thickness reduction and trabecular microarchitecture reductions were evident as early as 2 weeks, primarily characterized by a decrease in trabecular number and an increase in separation. These long-term structural impairments emphasize the chronic impact of irradiation on skeletal health.

Osteoclast numbers, similar to monocytes, decreased immediately following irradiation, indicating high sensitivity to irradiation-induced cell death [42, 43]. Indeed, no alteration was observed in the bone area covered by osteoclasts, indicating that the osteoclasts became larger and continued to cover the same area. Indeed, elevated CTX-1 serum levels (a marker of bone resorption) were observed 2- and 6-weeks post-irradiation and HSCT. *Rankl* and *Opg* gene expression and *Rankl/Opg* ratio in the bone marrow were not altered, but the semi-quantitative microarray showed a reduction in serum OPG 2 weeks post-irradiation, indicating a boost in osteoclast activity and emphasizing the ongoing dysregulation of osteoclast activity. In addition, *Rank* gene expression was borderline elevated in bone marrow both 1 day and 2 weeks post-irradiation. The increase in CTX-I and *Rank* indicates a positive regulation of osteoclast potential, which may translate to the reduced skeletal parameters, even though no effect was visible in the number of osteoclasts.

Osteoblast numbers and the surface covering bone area declined 2 weeks post-irradiation. Furthermore, the increase in bone marrow adipocytes indicates a shift in mesenchymal differentiation from an osteogenic to adipogenic lineage [44–46], likely impairing bone regeneration following irradiation-induced damage [47]. This was consistent with reduced serum PINP levels (a marker of bone formation) and RUNX2, a key regulator of osteoblast differentiation. The mechanisms driving adipocyte induction and their impact on osteoblast formation in this context remain poorly understood.

A critical observation was the sustained presence of apoptotic cells in the bone marrow and bone tissues up to 12 weeks post-irradiation. TUNEL-positive cells and elevated expression of the pro-apoptotic gene *Bax* persisted, indicating prolonged apoptosis and insufficient efferocytosis. This was accompanied by increased *Tgf-β1* gene expression in the bone marrow during the first 2weeks post-irradiation and HSCT. TGF-β1, which stimulates osteoclasts, is one of the most abundant cytokines in the bone matrix [48]. TGF-β1 is released during bone resorption [49], known to be induced by radiation [50, 51] and during efferocytosis [52]. Our experiments did not observe significant changes in cortical bone *Tgf-β1* expression and only limited increases in serum TGF-β1 levels, suggesting a localized regulatory effect confined to the bone marrow.

In the acute irradiation approach, it was evident that lymphocytes are the most radiosensitive cells, rapidly undergoing apoptosis. Macrophages were affected within the first 2 days, while neutrophils showed only a limited response. In the osteoclast cultivation assay, prolonged exposure to apoptotic cells did not affect the number or morphological parameters of osteoclasts. Apoptotic bone lining cells and osteoblasts from microfractures are known to stimulate osteoclasts by releasing cytokines and TGF-β1 [53]. In contrast, it is well established that macrophages engulfing apoptotic cells can produce TGF-β1 [54–56]. Interestingly, in our in vitro experiments, TGF-β1 levels were elevated in the supernatant from murine bone marrow-derived monocytes and pre-osteoclasts with apoptotic cells compared to cultures without apoptotic stimuli. Our findings suggest that apoptotic stimuli during osteoclast differentiation can sustain TGF-β1 production, which is likely dependent on efferocytosis. This may contribute to Tgf-β1 expression in the bone marrow, potentially influencing bone remodeling.

Indeed, our experiments observed elevated TRAP5b levels in the supernatant of osteoclast differentiation as well as in gene expression of the corresponding gene, *Acp5*, in the presence of apoptotic cells, compared to cultures without apoptotic stimuli. A similar pattern was also observed in the gene expression of *Nfatc1* and *Rank*, both of which are regulated by TGF-β1 [57–59]. While higher TRAP5b activity and elevated expression of *Nfatc1* and *Rank* are typically associated with increased osteoclast numbers in vitro, their primary roles involve osteoclast activation and may facilitate bone resorption.

To further elucidate the apoptotic stimuli, we compared them to TGF-β1 stimulation of bone marrow monocytes. With a limited dose of TGF-β1, we observed a similar phenotype to that of the apoptotic stimuli. This effect could be partly blocked using a TGF-β1 inhibitor. A similar type of partial blockade was also observed in the apoptotic stimulation. Therefore, in the in vitro cultivation of bone marrow macrophages towards osteoclasts, apoptotic cells appear to mediate their effect, at least in part, via TGF-β1.

Pre-osteoclasts and smaller osteoclasts rapidly engulfed apoptotic cells within 60 min, demonstrating efferocytosis activity, though this did not influence the number of mature osteoclasts. Indeed, previous studies have shown that osteoclasts activated as antigen-presenting cells maintain their bone-resorbing capacity [17]. The role of osteoclasts in efferocytosis was first described by Harre et al., who demonstrated that human osteoclasts can engulf apoptotic cells and express genes associated with the efferocytosis machinery [19]. The efferocytosis in bone was further described by Batoon et al., who demonstrated how altering the apoptotic capacity in mice influences bone tissue [20, 22]. Our findings demonstrate that murine bone marrow-derived pre-osteoclasts display efferocytosis capacity. In contrast, this ability was diminished in fully matured osteoclasts. Elevated *Tgf-β1* expression during in vitro cultivation, secreted during efferocytosis, modulates osteoclast activity and influences the bone microenvironment. The continued presence of apoptotic cells after irradiation likely stimulates Tgf-β1 in the bone marrow, which contributes to the local stimulation of bone-resorbing osteoclasts. Indeed, the borderline elevation of *Rank* expression further emphasizes the connection through the induction of TGF-β1 local stimulation within the bone marrow.

This study has several limitations. The use of a single inbred mouse strain (female C57BL/6) without variation in age or sex limits the generalizability of the findings but also reduces variability due to limitations of immune activation. While murine models offer mechanistic insight, their clinical relevance remains limited due to human biological variability, including age, underlying disease, and treatment differences that affect bone outcomes post-HSCT. Finally, although prolonged apoptosis and increased TGF-β1 expression were observed, the underlying mechanisms driving impaired bone regeneration, particularly the role of efferocytosis, have yet to be fully elucidated and require further investigation.

While irradiation does not cause immediate bone loss, it results in a continuous decline in both cortical and trabecular bone, starting 2 weeks post-irradiation and continuing throughout the 12-week study duration. Promoting efficient efferocytosis in the bone could help mitigate these effects and provide a therapeutic strategy to improve skeletal health.

## Supplementary information

Supplementary figure

## Data Availability

Supplementary information is available on the Cell Death & Disease website at the end of the article, just before the references. The data supporting the findings of this study are deposited in the FigShare repository 10.6084/m9.figshare.30814670.
