# Supplementary material for: Disrupted bone microenvironment and immune recovery following total body irradiation in a murine model
Source: Cell Death Dis. 2025 Dec 13;17(1):122. doi: 10.1038/s41419-025-08303-7 (PMC12847818; doi:10.1038/s41419-025-08303-7)
Supplement: Supplementary file 1 — Supplementary figure [file 41419_2025_8303_MOESM1_ESM.pdf]

Sup fig 1

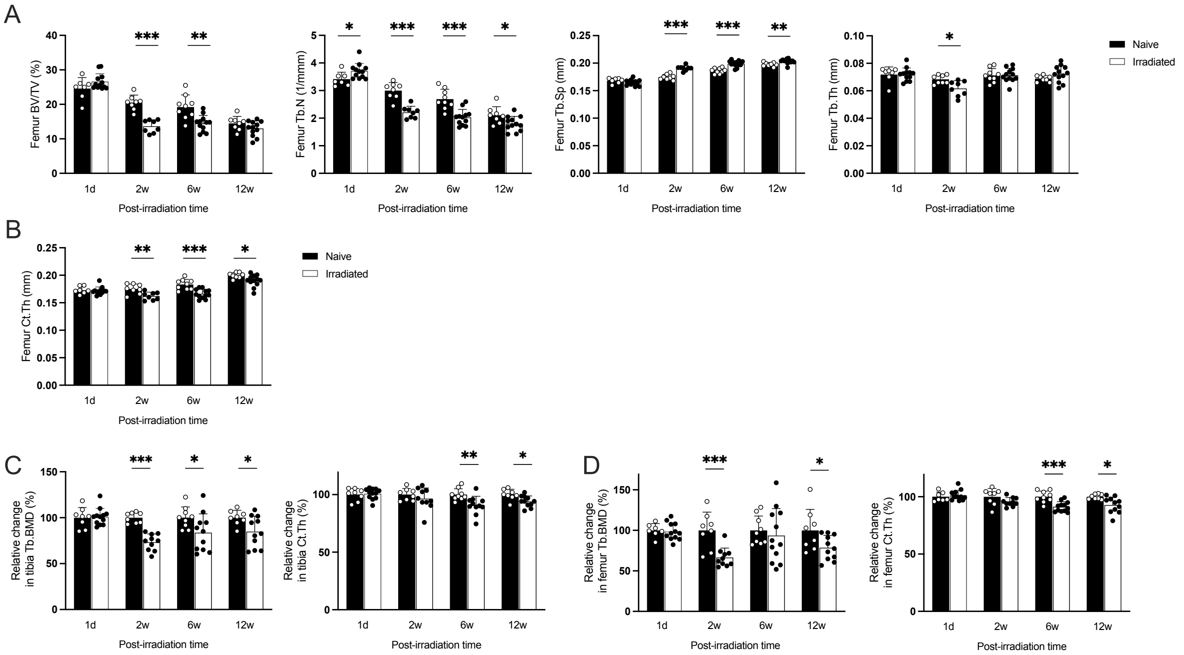

Fig. S1: Computer tomograph analysis of tibia and femur.

A. Micro-computed tomography (µCT) analysis of trabecular femur bone. Bone volume per total volume (Tb. BV/TV), trabecular number (Tb.N), trabecular separation (Tb.Sp), and trabecular thickness (Tb.Th) in absolute number. **B**. µCT of femoral cortical thickness (Ct.Th) in absolute number. **C.** Relative changes in trabecular volumetric bone mineral density (Tb. BMD) and cortical bone thickness (Ct. Th.) measured in the tibia by Peripheral Quantitative Computed Tomography (pQCT). **C**. Trabecular volumetric BMD and cortical bone thickness measured in the femur by pQCT. Statistical analysis was performed using the Student's t-test to assess differences between the irradiated and control mice at each time point. Sample sizes ranged from n=8 to 12. Data are presented as mean ± SD. Significance levels are indicated as *P < 0.05, **P < 0.01, ***P < 0.001.

Sup fig 2

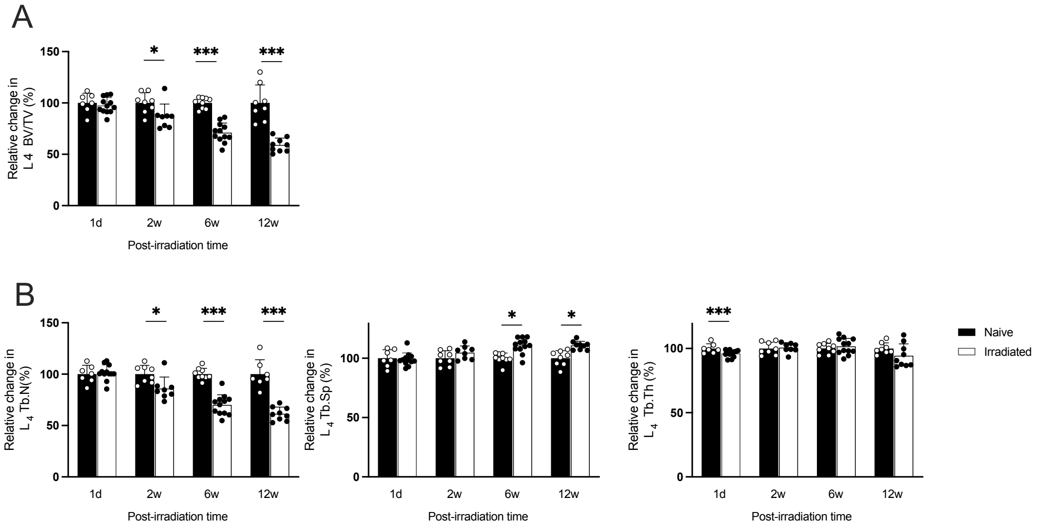

Fig. S2: High-resolution microcomputed tomography (µCT) analysis of L4 vertebrae.
A. Relative change in micro-computed tomography (µCT) analysis of trabecular bone volume per total volume (Tb. BV/TV) in lumbar vertebra 4 (L4). **B**. Relative change in L4 Trabecular Number (Tb.N), Trabecular Separation (Tb.Sp), and Trabecular Thickness (Tb.Th). Statistical analysis was performed using the Student's t-test to assess differences between the irradiated bone marrow transplanted and control mice at each time point. Sample sizes ranged from n=8 to 12. Data are presented as mean ± SD. Significance levels are indicated as *P < 0.05, **P < 0.01, ***P < 0.001.

Sup fig 3

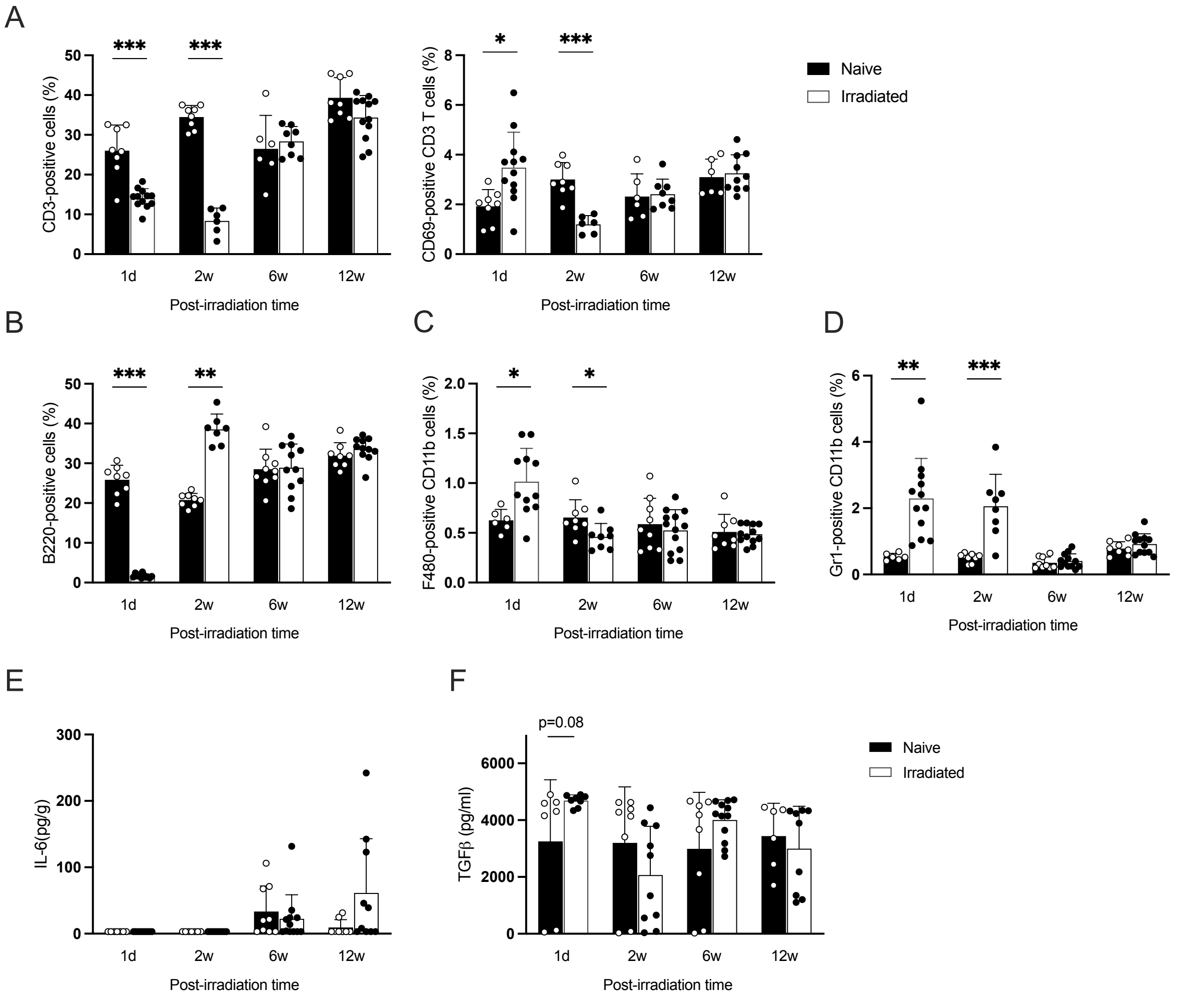

Fig. S3: Flow cytometric analysis of the spleen cell population in irradiated and bone marrow transplanted mice.

A. Relative change in CD3$^+$ T cells gated on the CD19$^-$ cell population. **B**. Relative change in B220$^+$ B cells gated on the CD3$^-$ cell population. **C**. Relative change in F480$^+$ monocytic subset gated from the CD3$^-$CD11b$^+$ population. **D**. Relative change in Gr1$^+$ neutrophil cells gated from the CD3$^-$CD11b$^+$ population. **E**. IL-6 and **F**. TGF-β1 protein levels were quantified in the irradiated and HSCT mice serum. **G**. Analysis of relative gene expression of *RANKL and OPG* genes in bone marrow, as well as the ratio between RANKL/OPG. Statistical analysis was performed using the Student's t-test to assess differences between the irradiated bone marrow transplanted mice and control mice at each time point. Data are presented as mean ± SD. Significance levels are indicated as *P < 0.05, **P < 0.01, ***P < 0.001.

Sup fig 4

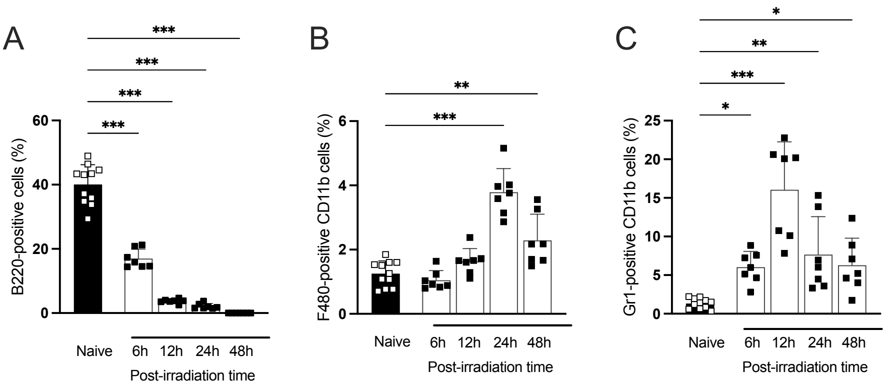

Fig. S4: Flow cytometric analysis of the spleen cell population in irradiated and bone marrow transplanted mice.

A. Relative change in $B220^{+}$ B cells gated on the $CD3^{-}$ cell population. **C**. Relative change in $F480^{+}$ monocytic subset gated from the $CD3^{-}CD11^{+}$ population. **D**. Relative change in Gr1+ neutrophil cells gated from the $CD3^{-}CD11^{+}$ population. Statistical analysis involved a one-way ANOVA followed by the Dunn–Sidák post-hoc test. Data are presented as mean ± SD, and significance is indicated as *P < 0.05, **P < 0.01, ***P < 0.001 compared to the control live population.

Sup fig 5

A

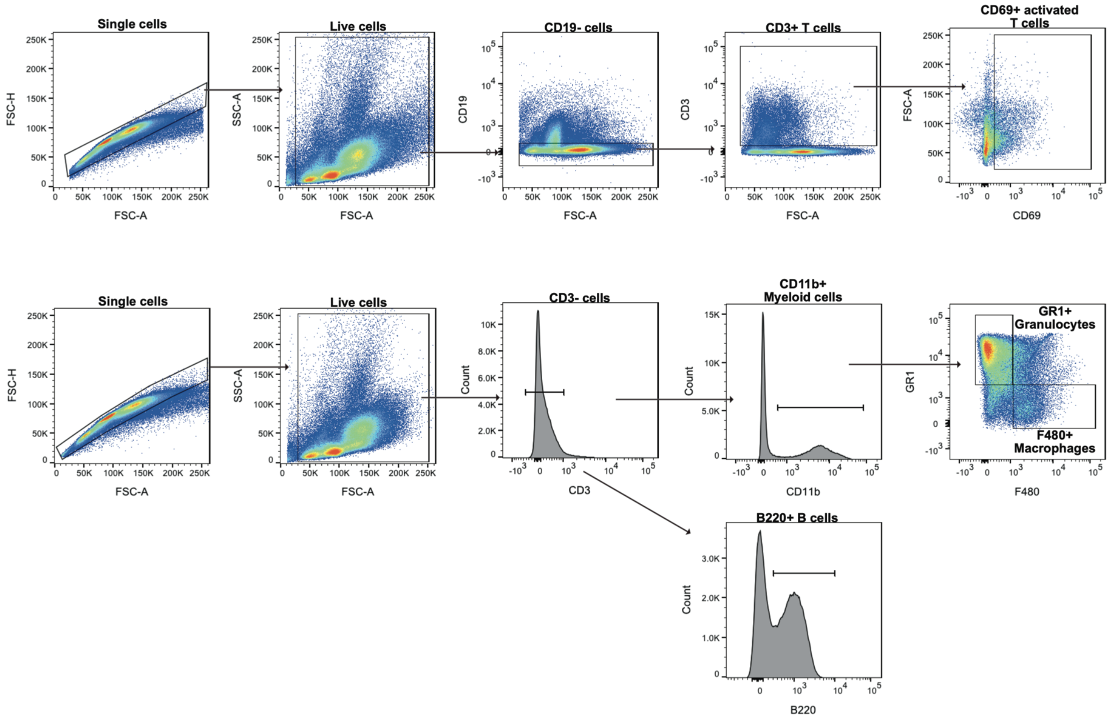

B

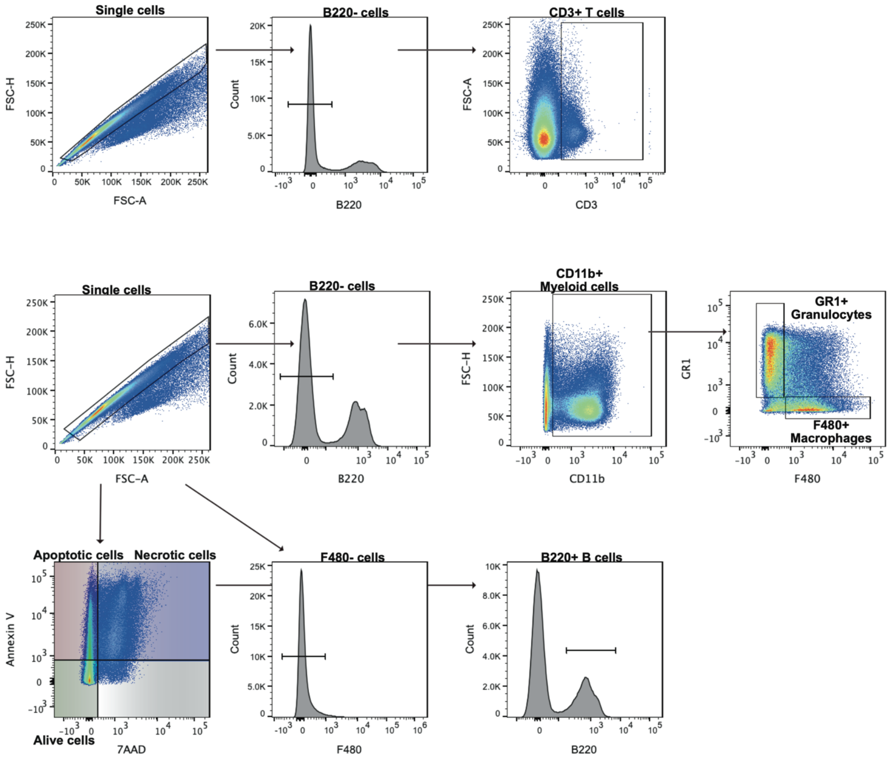

Fig. S5: Representative gating strategies for the bone marrow and spleen cell populations.

A. Cells from bone marrow were gated for forward scatter height vs. forward scatter area (FSC-H/FSC-A) before identification of total T cells ($CD19^{-}CD3^{+}$), activated T cells ($CD19^{-}CD3^{+}CD69^{+}$), granulocytes ($CD3^{-}CD11b^{+}Gr1^{+}$), macrophages ($CD3^{-}CD11b^{+}F4/80^{+}$), and pan B cells ($CD3^{-}B220^{+}$). **B**. Cells from bone marrow were gated for forward scatter height vs. forward scatter area (FSC-H/FSC-A) before identification of total T cells ($B220^{-}CD3^{+}$), granulocytes ($B220^{-}CD11b^{+}Gr1^{+}$), macrophages ($B220^{-}CD11b^{+}F4/80^{+}$), and B cells ($F480^{-}B220^{+}$). Cells were considered based on staining patterns: apoptotic cells were identified as Annexin V (Annexin V^{+}), necrotic cells as both Annexin V and 7-AAD (Annexin $V^{+}7AAD^{+}$), live cells as negative for both Annexin V and 7-AAD (Annexin $V^{-}7AAD^{-}$), and cell fragments or debris were characterized by high 7-AAD density.

Sup fig 6

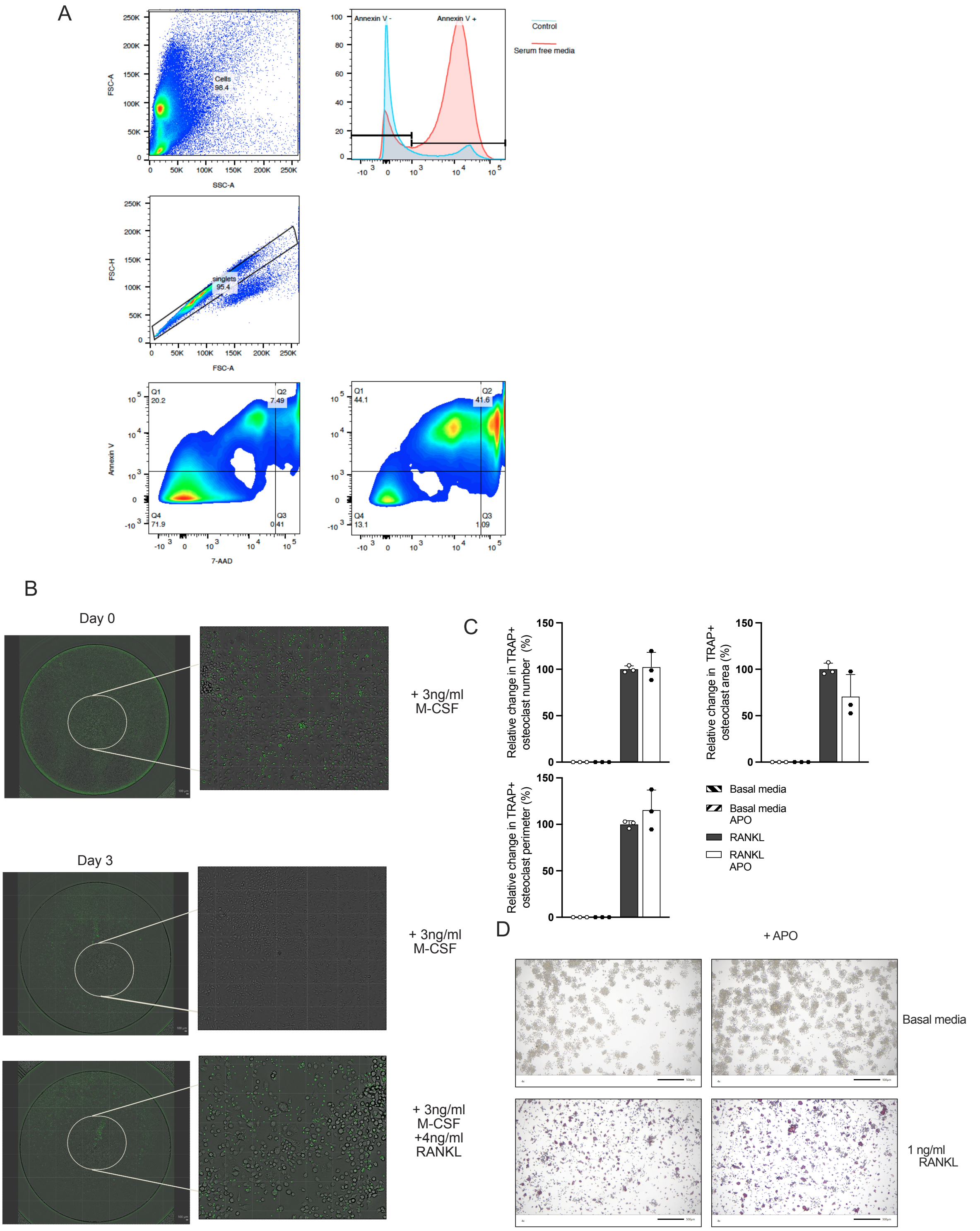

Fig. S6: Phagocytosis assay.

A. Representative flow cytometry gating strategy for identifying cell populations to validate thymocyte apoptotic ability for the phagocytosis assay. **B**. Validation of the long-term phagocytic capacity of osteoclasts using bone marrow macrophages as positive controls for internalizing FITC-conjugated apoptotic cells. **C**. Relative changes in the RAW 264.7 cells number, area, and perimeter of multinucleated TRAP+ cells after 3 days of differentiation, with

or without apoptotic cells. **D**. Representative images of osteoclast differentiation after 3 days of stimulation with or without the presence of apoptotic cells. Sample sizes n=3.

Sup fig 7

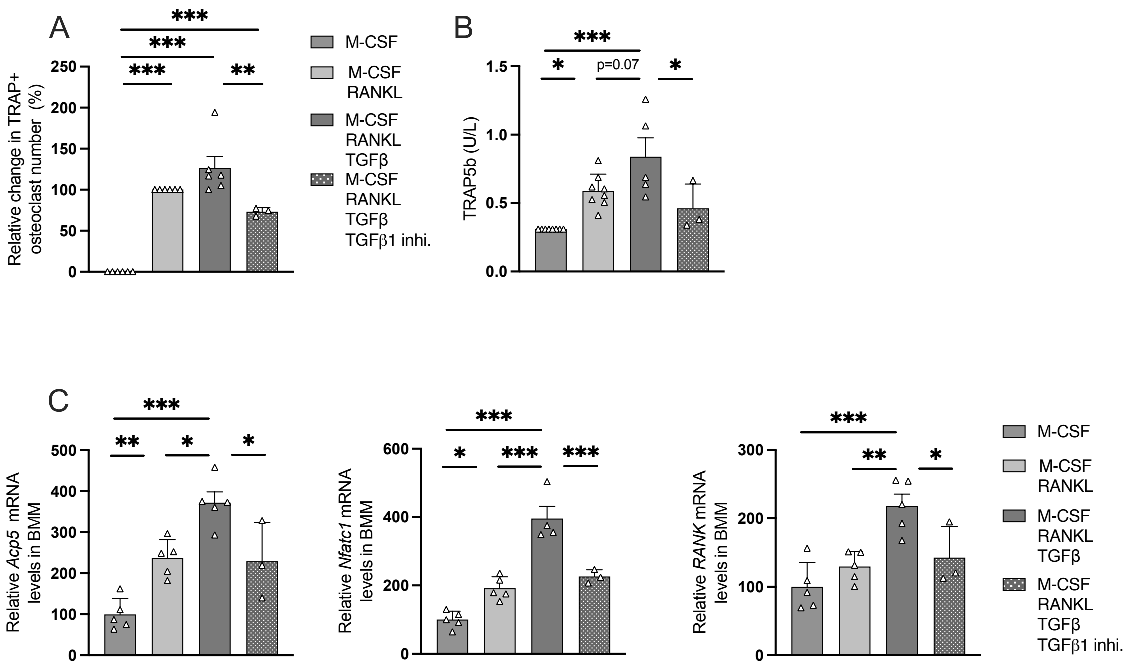

Fig. S7: TGF-β1 did not influence the number of osteoclasts but expression of genes and TRAP5b

A. Relative difference changes in the number of multinucleated TRAP+ cells after 3 days of differentiation, with or without apoptotic TGF-β1 and TGF-β1 inhibitor. **B.** TRAP5b protein expression in the supernatant of osteoclast differentiation. **C.** Analysis of relative gene expression of *Acp5, Nfatc1*, and *Rank* gene. Statistical analysis involved a one-way ANOVA followed by Tukey's multiple post-hoc test. Data are presented as mean ± SD, and significance is indicated as *P < 0.05, **P < 0.01, ***P < 0.001 (n=3-6)

	Mean pixel density		
**Serum marker**	**Control**	**2w**	**12w**
**Adiponectin/Acrp30**	16751	25082	28711
**BAFF/BLyS/TNFSF13B**	10250	9321	29887
**C-Reactive Protein/CRP**	26830	31668	28801
**CCL11/Eotaxin**	8375	11935	8693
**CCL21/6Ckine**	22360	33290	35033
**CD14**	7935	24157	14496
**Complement Component C5**	8843	16344	13414
**CX3CL1/Fractalkine**	25061	36294	48671
**CXCL16**	20440	34220	40480
**Cystatin C**	21297	34783	7935
**DPPIV/CD26**	8182	12269	8419
**Endostatin**	31704	40265	41208
**Flt-3 Ligand**	8391	13124	12728
**Gas 6**	12630	22819	3569
**IGFBP-5**	9055	7939	7934
**IL-6**	7935	7935	8380
**Leptin**	8095	7892	7943
**MMP-2**	23824	28701	35774
**MMP-3**	20454	24611	29287
**Osteoprotegerin**	9013	11152	2280
**Proliferin**	7935	9892	9983
**Resistin**	19123	19190	19027
**TNF-alpha**	7935	7958	7952

Table S1 The mean pixel density of each serum marker

Changes in serum marker expression levels were determined based on the normalized pixel density.
